# Ternary Blends of PLA with ATEC and TMC-200 as Medical-Grade Biodegradable Monofilaments for FDM 3D-Printing Applications

**DOI:** 10.3390/polym17212926

**Published:** 2025-10-31

**Authors:** Manasanan Namhongsa, Tanyaluck Mekpothi, Kittisak Yarungsee, Donraporn Daranarong, Gareth M. Ross, Sukunya Ross, Winita Punyodom

**Affiliations:** 1 Bioplastics Production Laboratory for Medical Applications, Faculty of Science, Chiang Mai University, Chiang Mai 50200, Thailand; yeastmanas@gmail.com; 2 Center of Excellence in Materials Science and Technology, Chiang Mai University, Chiang Mai 50200, Thailand; 3 Office of Research Administration, Chiang Mai University, Chiang Mai 50200, Thailand; tanyaluck.me@cmu.ac.th; 4 Department of Chemistry, Faculty of Science, Chiang Mai University, Chiang Mai 50200, Thailand; kittisak.yr@gmail.com; 5 Multidisciplinary Research Institute, Chiang Mai University, Chiang Mai 50200, Thailand; d.daranarong@gmail.com; 6 Center of Excellence in Biomaterials, Department of Chemistry, Faculty of Science, Naresuan University, Phitsanulok 65000, Thailand; gareth@nu.ac.th (G.M.R.); sukunyaj@nu.ac.th (S.R.)

**Keywords:** poly(L-lactide) monofilament, fused deposition modeling, nucleating agent, plasticizer

## Abstract

Poly(L-lactide) (PLA) is a promising biopolymer for biomedical applications due to its biodegradability and biocompatibility; however, its brittleness restricts its use in fused deposition modeling (FDM). To overcome this limitation, flexible PLA monofilaments with enhanced mechanical performance and printability were developed. In this study, PLA was melt-blended with acetyl triethyl citrate (ATEC, 1.0–5.0 wt%) as a plasticizer and zinc phenyl phosphonate (TMC-200, 0.3 wt%) as a nucleating agent. It was found that the PLA with 3.0 wt% ATEC (PLA/A) exhibited the greatest flexibility, while the addition of TMC-200 further improved tensile strength and ductility. Specifically, the ternary blend of PLA/TMC-200/ATEC (PLA/T/A) exhibited a synergistic effect, achieving superior mechanical properties (tensile strength: 35.0 MPa, elongation at break: 232.0%, compared to 12.1% for pure PLA) and raising the degree of crystallinity (*X*_c_) from 4.7% to 45.0%. Monofilaments (1.70 ± 0.05 mm) fabricated from PLA/T/A exhibited smooth surfaces, balanced mechanical performance, and excellent cytocompatibility (over 99% cell viability in L929 fibroblasts). Moreover, FDM-printed specimens retained enhanced mechanical and thermal performance, demonstrating material stability after processing. Shelf-life testing further confirmed the structural integrity of PLA/T/A monofilament after 8 weeks at 50 °C. Overall, PLA/T/A provides an effective strategy for producing high-performance, medical-grade PLA monofilaments with improved toughness, printability, and biocompatibility, enabling their application in biomedical 3D printing.

## 1. Introduction

Three-dimensional (3D) printing, also known as additive manufacturing (AM), is widely used in tissue engineering, biomedical devices, and 3D scaffold development, owing to its capability to rapidly and cost-effectively produce complex geometries and intricate architectures [1,2]. Current 3D printing techniques include fused deposition modeling (FDM), inkjet printing (IJP), stereolithography (SLA), powder bed fusion (PBF), laminated object manufacturing (LOM), direct energy deposition (DED), direct write, and 3D bio-plotting [3,4]. Among these, FDM is the most commonly employed method because of its simplicity, speed, and cost-efficiency. Poly(L-lactide) (PLA) is a particularly attractive material for FDM due to its biodegradability, biocompatibility, and favorable mechanical strength, as well as its derivation from renewable resources [4,5]. However, the inherent brittleness and low toughness of PLA restrict its broader applications, thereby driving extensive efforts to enhance its mechanical performance and ensure its suitability for large-scale production in the FDM industry [6,7,8,9,10].

Improving the flexibility and printability of PLA remains a major challenge. Several strategies, including copolymerization, surface modification, polymer blending, and filler incorporation, have been studied to mitigate PLA’s limitations [11,12,13,14,15]. Currently, researchers have focused on reinforcing materials—such as fibers (natural or synthetic) and powders (ceramic, metallic, carbon-based, and inorganic nucleating agents)—to overcome the mechanical and thermal limitations of neat PLA in FDM applications [16]. However, these reinforcements often reduce filament flexibility and increase brittleness, leading to breakage and poor printability. Weak interfacial adhesion may cause delamination, while uneven filler dispersion can result in nozzle clogging and rough surfaces. High filler loadings also increase melt viscosity, limiting extrusion flow and processing efficiency [17].

At present, an effective approach to enhance the processability and cost-efficiency of 3D printing monofilament materials is the incorporation of plasticizers such as acetyl tributyl citrate (ATBC), acetyl triethyl citrate (ATEC), triethyl citrate (TEC), triacetine (TA), tributyl citrate (TBC) and polyethylene glycol (PEG), which improve flexibility and facilitate monofilament extrusion while preserving biodegradability, thus providing a cost-effective solution without the necessity of synthesizing new polymers [5,11,18,19,20,21,22,23,24,25]. Several studies have investigated the selection of suitable plasticizers and the optimization of their concentrations to enhance the performance of PLA monofilaments for FDM 3D printing. Kumar et al. [26] found that the incorporation of PEG significantly improved the tensile strength and printability of PLA, although the elongation at break slightly decreased with 1, 3, and 5 wt% PEG loading. The addition of 5 wt% PEG to the PLA 3D printed samples was found to be optimal, yielding a maximum tensile strength of 32.06 MPa and an elongation at break of 2.29 mm/mm. Gao et al. [27] obtained a similar result of poor tensile strength with an increase in the PEG content in PLA. The tensile strength and tensile modulus were in the ranges of 39–55 MPa and 1000–1400 MPa, respectively. Additionally, Carlier et al. [23] compared the plasticizing effects of various plasticizers (PEG, TA, ATEC, and TEC) at 10 wt% in PLA for fused deposition modeling. They found that ATEC exhibited the most pronounced plasticizing effect, leading to the greatest reduction in both Young’s modulus and tensile strength compared to the other plasticizers. In contrast, PEG 400 showed no significant influence on Young’s modulus and only a slight reduction in tensile strength. Lui et al. [25] compared the plasticizing effect of different citrates (TEC, ATEC, TBC, and ATBC), reporting a similar trend in their study on poly(vinyl chloride) (PVC). The tensile results revealed that the acetylated citrates, ATEC and ATBC, exhibited superior plasticizing efficiency compared to their non-acetylated counterpart. Thus, ATEC, in particular, has been identified as a highly miscible and biocompatible plasticizer that effectively enhances PLA flexibility, making it suitable for biomedical and food-contact applications [20], whereas, plasticization alone often reduces stiffness and dimensional stability, which compromises the mechanical performance of printed parts [22].

To address these issues, nucleating agents have been introduced to accelerate crystallization and enhance mechanical properties, particularly by improving tensile strength compared to the use of a plasticizer alone. The TMC series nucleating agents (TMC-200, TMC-300, and TMC-306) are organic nucleating agents that can significantly enhance the crystallization rate and mechanical properties of PLA. These agents—including small organic molecules, organic salts, and polymers—also help prevent agglomeration during fused filament fabrication [17,28,29]. Shi Xu et al. [30] also reported that TMC-200, along with other TMC series nucleating agents (TMC-300 and TMC-306), could achieve effective impact toughening of PLA/PEG blends for packaging film materials. Among these, TMC-200 was superior, simultaneously improving the crystallinity, stiffness, and heat resistance of PLA, even at very low additive concentrations (<0.5 wt%), compared to other nucleants. Another recent study confirmed that in PLA blends such as PLA/PCL, TMC-200 exhibited supernucleation behavior and sometimes outperformed other TMC derivatives, although the resulting improvements in mechanical properties were influenced by processing parameters like molding temperature [28]. In addition, Gao et al. [29] demonstrated that combining plasticizers (e.g., PEG) with nucleating agents such as TMC-306 can synergistically modify the crystallization kinetics of PLA during fused filament fabrication, thereby influencing the crystallinity and final properties of the printed parts. They reported that the crystallinity increased from approximately 8% for both neat PLA and PLA/TMC samples to about 18% for the PEG-containing formulations; however, the mechanical properties of the filaments and printed parts were adversely affected.

Indeed, studies combining nucleating agents with plasticizers or fillers to simultaneously improve toughness and crystallinity remain limited, especially for medical-grade FDM filaments. Cytotoxicity and shelf-life assessments are often lacking. While previous works have explored individual additive effects, systematic evaluation of their synergistic influence on biocompatible PLA for medical 3D printing is needed. Combining TMC-200 with plasticizers such as ATEC could enhance toughness, strength, and printability, addressing the critical gap of achieving both high mechanical performance and cytocompatibility [31].

In this study, PLA was first plasticized with ATEC at varying concentrations (1.0, 1.5, 3.0, and 5.0 wt%) to optimize flexibility and processability for medical FDM applications. PLA and ATEC were blended via reactive melt blending, and the effects of plasticization on mechanical properties, thermal behavior, and inherent viscosity were systematically evaluated. The optimal formulation was further modified with 0.3 wt% TMC-200 to enhance the mechanical properties of PLA. Although previous studies have examined the individual effects of plasticizers and nucleating agents on PLA, this investigation represents the first comprehensive evaluation of their combined synergistic influence on mechanical, thermal, rheological, and crystallization behavior, as well as polarized optical microscopy (POM). Monofilaments were fabricated with precise diameters (1.70 ± 0.05 mm) and further characterized for surface morphology, mechanical performance, thermal properties, cytotoxicity, and shelf-life evaluation. Finally, the extruded monofilaments were further tested in FDM printing, where the enhanced mechanical and thermal properties were retained in the printed specimens, confirming their printability and suitability for medical 3D printing applications.

## 2. Materials and Methods

### 2.1. Material

In this work, medical-grade L-lactide (LL) monomer was provided by the Bioplastics Production Laboratory for Medical Applications (ISO 13485:2016 TÜV SÜD America Accredited Laboratory, Chiang Mai University, Chiang Mai, Thailand). Triethyl O-acetylcitrate (ATEC, >97% with ${\bar{M}}_{w}$ ~318.32 g/mol) was supplied by Tokyo Chemical Industry (Tokyo, Japan). Zinc phenyl phosphonate (TMC-200) was purchased from the Shanxi Provincial Institute of Chemical Industry, China. Chloroform (CHCl_3_, ≥98%) was purchased from Sigma-Aldrich (St. Louis, MO, USA). 

### 2.2. Preparation of PLA Pellets

**Figure 1:**
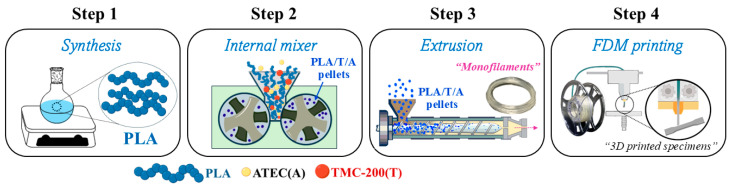
Schematic representation of the processing steps for PLA/TMC-200/ATEC (PLA/T/A) ternary blends: synthesis, melt mixing, extrusion, and FDM printing.

The synthesis of PLA was conducted in step 1 of Figure 1. In this method, PLA pellets were prepared via bulk ring-opening polymerization (ROP) using liquid tin(II) *n*-butoxide (Sn(*On*Bu)_2_) as an initiator. Firstly, the LL monomer was accurately weighed and simultaneously added to a 500 mL round-bottom flask equipped with a magnetic stirring bar under a dry nitrogen (N_2_) atmosphere. Subsequently, bulk polymerization of PLA polymers was carried out in an oil bath at 120 °C for 72 h under vacuum. The reaction flask was then allowed to cool to room temperature. The crude PLA was purified by cutting and grinding into small pieces to increase its surface area and then drying to constant weight in a vacuum oven at 80 °C to remove any trace amounts of residual monomers and moisture. Finally, it was vacuum sealed and stored in the refrigerator at −20 °C for future use.

### 2.3. Preparation of PLA Blend Pellets by Internal Mixer

PLA pellets were melt-blended with additives using an internal mixer (HAAKE™ Rheomix OS Lab Mixer, Thermo Fisher Scientific, Karlsruhe, Germany), as depicted in step 2 of Figure 1. To prevent moisture-induced degradation, PLA was pre-dried at 80 °C for 24 h. The additives ATEC (at 1.0, 1.5, 3.0, and 5.0 wt%) and TMC-200 (at 0.3 wt%) were individually incorporated into PLA and melt-blended at 180 °C for 5 min at a screw speed of 50 rpm. The resulting homogeneous compounds were then ground into pellets. Subsequently, the PLA/T/A blend containing 3.0 wt% ATEC and 0.3 wt% TMC-200 was subjected to an additional melt-blending step under the same conditions. The preparation details of the PLA blends—PLA/ATEC (PLA/A), PLA/TMC-200 (PLA/T), and PLA/TMC-200/ATEC (PLA/T/A)—are summarized in Table 1.

### 2.4. Fabrication of PLA, PLA/A, PLA/T and PLA/T/A Monofilaments by Melt Extrusion

PLA and the selected PLA-based blends—PLA/A (containing 3.0 wt% ATEC), PLA/T (containing 0.3 wt% TMC-200), and PLA/T/A (containing 3.0 wt% ATEC and 0.3 wt% TMC-200), as described in Section 3.1 and Section 3.2—were oven-dried at 50 °C for over 24 h before being used to extrude monofilaments. The single-screw extruder featured seven temperature zones (Collin Lab & Pilot Solutions GmbH, Ebersberg, Germany), set sequentially as follows: 40 °C, 160 °C, 160 °C, 160 °C, 160 °C, 155 °C, and 155 °C, respectively. The screw speed was maintained at 50 rpm. The resulting PLA, PLA/A, PLA/T, and PLA/T/A monofilaments had diameters ranging from 1.70 mm to 1.80 mm, were coiled onto spools, and stored in zip-lock bags before characterization, as shown in step 3 of Figure 1.

### 2.5. Fabrication of Printed PLA, PLA/A, PLA/T and PLA/T/A Specimens by FDM 3D Printing

The monofilaments prepared as described in Section 2.4 were subsequently processed into printed specimens using FDM 3D printing (Bambu Lab, Shenzhen, China), as depicted in step 4 of Figure 1. Firstly, a 3D model for a tensile test was designed using computer-aided (3D) design (Bambu Lab A1, version 3.1.0), as per the ASTM standards (ASTM D638 Type I: tensile properties of plastics) [32], and subsequently was sliced using slicing software (version 3.1.0; Bambu Lab, Shenzhen, China) and converted to stereolithography (STL) format. The specimens were printed using FDM 3D printing with a 0.4 mm nozzle. The nozzle temperature was set between 160 °C and 240 °C, with layer infill and outer wall printing speeds of 105 mm/s and 200 mm/s, respectively. The layer thickness was 0.16 mm, and the platform temperature was maintained at 80 °C. The sparse infill density of the 3D-printed specimens was set at 15% in a grid pattern.

### 2.6. Characterization of PLA Blend Pellets, Monofilaments, and Printed Specimens

#### 2.6.1. Morphology

The surface morphologies of PLA, PLA/A, PLA/T, and PLA/T/A monofilaments were characterized using a scanning electron microscope (SEM; JEOL 5910 LV, JEOL Ltd., Tokyo, Japan). For sample preparation, the monofilaments were mounted onto metal stubs using carbon tabs and then sputter-coated with gold at an acceleration voltage of 15 kV.

#### 2.6.2. Chemical Functionalities

The chemical functionalities of PLA, PLA/A, PLA/T, and PLA/T/A monofilaments were investigated using an attenuated total reflectance (ATR-FTIR model, Nicolet^TM^ iS5, Thermo Scientific^TM^, Waltham, MA, USA). The FTIR spectra were recorded with a wavenumber range of 400 to 4000 cm^−1^.

#### 2.6.3. Thermal Properties

The thermal properties of PLA blended pellets, monofilaments, and printed specimens were determined by a differential scanning calorimeter (DSC; DSC 7, PerkinElmer Inc., Waltham, MA, USA). For each experiment, the samples were weighed between 5–10 mg and then sealed into a 50 mL aluminium pan. Measurements were performed at a heating rate of 10 °C/min from 0 °C to 220 °C in a nitrogen (N_2_) atmosphere. The transition temperatures (i.e., T_g_, T_c_, and T_m_) values and the degree of crystallinity (*X*_c_) were obtained from the second scan after eliminating the thermal history in the first scan.

The degradation temperature (T_d_) was determined using thermogravimetric analysis (TGA; TGA 7, PerkinElmer Inc., Waltham, MA, USA). For all measurements, 5–10 mg of the polymer sample was placed into a 50 mL aluminum pan and heated at a heating rate of 20 °C/min from 50 °C to 550 °C under a flowing nitrogen atmosphere.

#### 2.6.4. Crystallinity

The crystallization morphology of PLA blended pellets was investigated by X-ray diffraction (XRD) using a Rigaku MiniFlex 600 diffractometer (Rigaku Corporation, Tokyo, Japan) with a diffraction angle range (2θ) from 5° to 35° (Cu Kα, 1.54 Å).

#### 2.6.5. Mechanical Properties

The tensile properties of PLA blended pellets, monofilaments, and printed specimens were measured using a universal testing machine. Initially, all blended samples were first prepared by a solvent-casting method. The PLA blended pellets were vacuum-dried in an oven at 50 °C for over 24 h. The PLA blended pellets were individually dissolved in the CHCl_3_ solvent at concentrations of 10% *w*/*v*, with a magnetic stirrer for 24 h. After 24 h, the solution of PLA blended pellets was poured into a Petri dish for solvent casting. To evaporate the solvent, the samples were kept in a fume hood for approximately 24 h at room temperature. Furthermore, the films were dried in the vacuum oven at 50 °C for approximately 4 h to remove any residual solvent or moisture. For tensile tests, films of PLA blended pellets were cut into 10 × 50 mm pieces and clamped at each end with mechanical grips. The tensile test was performed at a strain rate of 10 mm/min using a 100 N load cell. Monofilament samples had an initial gauge length of 60 mm and were tested at a crosshead speed of 10 mm/min with a 1 kN load cell and bollard grips. Stress and strain data were calculated from load and displacement. The tensile tests of all films and monofilaments were performed using a universal mechanical testing machine (LS2.5, Lloyd Instruments/Ametek, Berwyn, PA, USA). For the printed specimens of PLA, PLA/A, PLA/T, and PLA/T/A, all specimens used for tensile testing were produced according to ASTM D638 Type I. The tensile tests of printed specimens were conducted at a strain rate of 10 mm/min with a 5 kN load cell, using a universal mechanical testing machine (Instron 5965, Instron Corp., Norwood, MA, USA). In all mechanical tests, data were collected from five specimens for each sample, and average values were reported.

#### 2.6.6. Polarized Optical Microscope (POM)

Crystalline morphology was analyzed via isothermal crystallization using a polarized optical microscope (BX53, Olympus Corporation, Tokyo, Japan). Briefly, thin films of PLA blended pellets were heated at 180 °C for 3 min and 130 °C for 30 min to erase thermal history, then cooled to room temperature and observed for crystallized structures.

#### 2.6.7. Melt Flow Index (MFI)

The melt flow index (MFI) measurements of PLA blended pellets were carried out by Tinius Olsen Extrusion Plastometer Melt Indexer (Model MP393, Tinius Olsen, Horsham, PA, USA) by applying a standard weight of 1.2 kg at a melting temperature of 180 °C. Then, the samples (10 g) were placed into a heated microcapillary, and the preheating time was set for 2 min. The measurement was extruded through a die with a cutting time of 30 s. The average melt quality through the standard microcapillary within 5 min was calculated using the mass method.

#### 2.6.8. Rheology Properties

The rheological behaviors of PLA blended pellets were studied using a rheometer (MCR 302e, Anton Paar, Graz, Austria) at time intervals. The samples were cut and placed between two 25 mm serrated parallel plates with a 1 mm gap. For strain sweep dynamic testing, the measurement was performed at 180 °C, and the shear strain amplitude was varied from 0 to 100%. The strain sweep was carried out at 1 Hz. To investigate the time sweep, the measurement was performed at a constant temperature of 180 °C for 60 min. The storage modulus (G′), loss modulus (G″), and complex viscosity (η*) were detected at a constant frequency of 1 Hz and a constant strain of 5%.

#### 2.6.9. Indirect In Vitro Cytotoxicity

The cytotoxicity of the PLA, PLA/A, PLA/T, and PLA/T/A monofilaments was studied by the MTT assay (3-(4,5-dimethyl-2-thiazolyl)-2,5-diphenyl-2H-tetrazoline bromide) based on ISO 10993-5 using the mouse fibroblast cell line (L929) [33]. Briefly, a cell suspension of 1 × 10^5^ cells/mL L929 in 10% serum (FBS)-containing Dulbecco’s Modified Eagle’s Medium (DMEM) complete medium was seeded into the 96-well plate. For the extraction solution, the monofilament samples of length 2 cm were cut into the 96-well plate and sterilized for 30 min under UV light. After that, 1 mL of DMEM completed medium was seeded into the 96-well plate on the samples. The 96-well plates were incubated at 37 ± 1 °C, 5 ± 0.1% CO_2_, and 95 ± 5% humidified atmosphere for 24 ± 2 h.

After incubation, the DMEM complete medium was removed. The mass-to-volume extraction ratio of 0.2 g/mL was used and then seeded into each well of the viable cells. Cells were then incubated for 24 ± 2 h. After an incubation period, the viable cells were stained with MTT at a concentration of 0.5 mg/mL in medium and incubated a further 2 h. Subsequently, the MTT solution was removed, and 100 µL dimethyl sulfoxide (DMSO) was added to each well and shaken for 10 min. The cell viability was determined by a microplate reader at 570 nm. The value of each sample was compared with the extracts of blank, negative-control, positive-control, and test specimens. The percentage of cell viability was calculated using the following equation;

Cell viability (%) = [(OD_s_)/(OD_c_)] × 100 (1)

where OD_s_ is the mean value of the measured optical density of the 100% extracts of the monofilament and OD_c_ is the mean value of the measured optical density of the 100% extracts of the blanks (incubated with culture medium without monofilaments).

#### 2.6.10. Shelf Life: Physical Appearance and Weight Retention

The assessment of storage temperature and shelf life on PLA and PLA/T/A monofilaments was explored using optical microscopy and a weight retention profile. All the monofilament samples were sealed in plastic and aluminum bags, which were then stored at 50 °C. For each temperature storage, the monofilaments were analyzed before storage and then after 2, 4, and 8 weeks. All the experiments were repeated three times under the same conditions, and the average values were reported. The physical appearances of monofilaments were observed on optical microscopy (OM; SZ650 series, Shanghai Drawell Scientific Instrument Co., Ltd., Shanghai, China). All experiments were captured and recorded using a digital camera 6.0 software. The samples were taken out and monitored each time. At the same time, the monofilament samples of length 20 cm were prepared and stored at 50 °C. All the monofilament samples were taken out and then weighed at various time intervals. The percentage weight retention was calculated as per the following equation:

Weight retention (%) = (W_t_/W_0_) × 100 (2)

where W_t_ is the weight of monofilaments at time t and W_0_ is the initial weight.

## 3. Results and Discussion

In developing PLA monofilaments for FDM-based medical applications, overcoming PLA’s inherent brittleness is essential to achieve the mechanical performance required for both filament fabrication and the resulting printed parts. Therefore, this work explored the synergistic effects of the plasticizer ATEC with varying concentrations (1.0, 1.5, 3.0, and 5.0 wt%) and the nucleating agent of TMC-200 (0.3 wt%) to identify optimal conditions for effective PLA blended monofilament fabrication. The selected formulations containing ATEC, TMC-200, and their combination (ATEC/TMC-200) were incorporated into the PLA matrix. The resulting monofilaments were then characterized in terms of surface morphology, mechanical properties, thermal behavior, shelf life, printability, and in vitro toxicity—marking a first-time exploration for FDM monofilament production.

### 3.1. Optimization and Characterization of PLA Blended with ATEC

PLA was initially blended with the plasticizer ATEC with varying concentrations (1.0, 1.5, 3.0, and 5.0 wt%) using internal mixers to ensure thorough blending. The resulting blends were designated as PLA/A (PLA with ATEC), as summarized in Table 1. After blending, the materials were cut into small pieces for subsequent characterization. Comprehensive testing was conducted to assess key functional characteristics, including mechanical properties (tensile strength, elongation at break, and tensile modulus), thermal properties, inherent viscosity, and melt flow index.

#### 3.1.1. Chemical Functionalities of PLA Blends with ATEC

**Figure 2:**
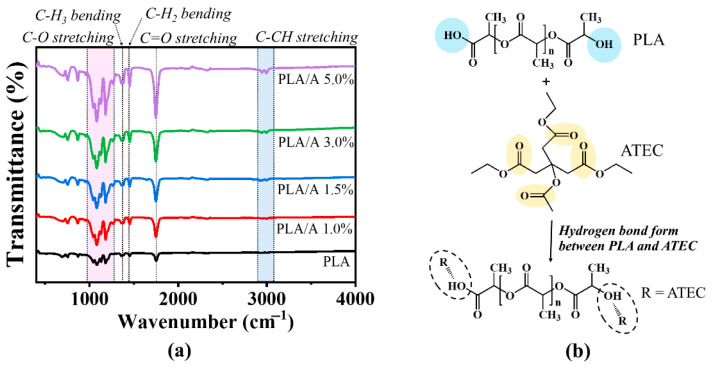
Structures and FTIR spectra of PLA and PLA/ATEC blends containing 1.0, 1.5, 3.0, and 5.0 wt% ATEC (A): (**a**) infrared spectra; (**b**) structural formula.

The main functional groups of PLA and PLA/A blends were identified by absorption bands at 1740–1750 cm^−1^ and 695–760 cm^−1^, corresponding to the C=O group (Figure 2a). In pure PLA, characteristic peaks were observed at 2997 cm^−1^ and 2947 cm^−1^ (asymmetric and symmetric stretching of CH_3_), 2879 cm^−1^ (C–H stretching), and 1362 cm^−1^ (C–H bending). Regarding the spectra of the plasticized blends, the characteristic peaks of all PLA samples were similar to those of pure PLA, indicating that no new chemical bonds were formed. However, increasing ATEC concentration (1.0–5.0 wt%) resulted in notable changes in peak intensity. In particular, the carbonyl absorption of PLA overlapped with that of ATEC, suggesting physical interactions between PLA and the plasticizer (Figure 2b) [24,34].

#### 3.1.2. Mechanical Properties of PLA Blends with ATEC

**Figure 3:**
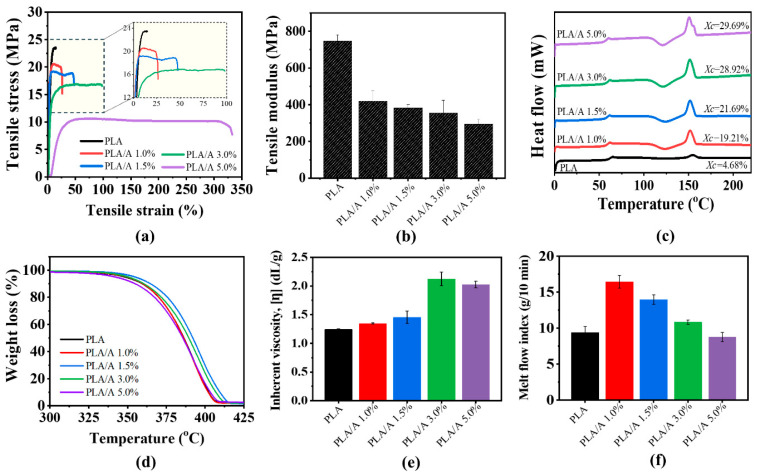
Characterization of PLA and PLA/A blends containing 1.0, 1.5, 3.0, and 5.0 wt% ATEC: (**a**) tensile stress–strain curves; (**b**) tensile modulus; (**c**) DSC thermograms (second heating); (**d**) TGA thermograms; (**e**) inherent viscosity; (**f**) melt flow index.

The mechanical properties of polymers are critical in defining their potential applications. To assess these properties, PLA and PLA/A blends were prepared using an internal mixer and subsequently fabricated into test films (Figure 3a,b, and Table 2). As illustrated, pure PLA exhibits a tensile strength of approximately 23 MPa and a very low elongation at break (~12%), confirming its brittle nature. With the addition of 1.0 wt% and 1.5 wt% ATEC, the tensile strength and modulus slightly decrease, while the elongation at break significantly increases to approximately 20–40%, indicating enhanced flexibility. At 3.0 wt% ATEC, a more noticeable reduction in tensile strength and modulus is observed, along with a further increase in elongation to about 60%. At 5.0 wt% ATEC, the tensile strength drops sharply to ~10 MPa, while the elongation at break increases markedly to ~70%, indicating a clear shift toward ductile behavior. These results demonstrate that ATEC functions as an effective plasticizer for PLA, with increasing content leading to a trade-off between tensile strength and flexibility [24].

#### 3.1.3. Thermal Properties of PLA Blends with ATEC

The thermal behavior of PLA and PLA/A blends was analyzed using DSC and TGA to evaluate the influence of plasticizer content on processability and performance in melt extrusion and FDM (Figure 3c,d, and Table 2) [35,36,37]. DSC analysis revealed that the incorporation of ATEC slightly decreased the glass transition temperature (T_g_), crystallization temperature (T_c_), and melting temperature (T_m_) across all blend samples, which is comparable to that of pure PLA (Figure 3c). As expected, PLA/A blends containing 1.0–3.0 wt% ATEC showed slight decreases in T_g_ and T_m_, attributed to molecular interactions—particularly hydrogen bonding—between PLA and ATEC, which enhance chain mobility [38]. At 5.0 wt% ATEC, excessive plasticizer caused two melting peaks, indicating varied crystal sizes or polymorphs within the blend. Accordingly, the degree of crystallinity (*X*_c_) increased markedly with ATEC content, from 4.68% for pure PLA to 19.21% (PLA/A 1.0 wt%), 21.69% (PLA/A 1.5 wt%), 28.92% (PLA/A 3.0 wt%), and 29.69% (PLA/A 5.0 wt%). This trend suggests that ATEC not only enhances chain mobility but also promotes ordering of PLA molecular chains, leading to higher crystallinity [23,38].

As illustrated in Figure 3d, TGA was used to evaluate the thermal stability and processing window of PLA and PLA/A blends, indicating the temperature at which the polymer can safely melt without thermal degradation (T_d_) [37]. Regarding the TGA curve, it was observed that all samples exhibited a single-step weight loss profile leading to complete decomposition (100%) with no residual remaining, suggesting good compatibility between PLA and the plasticizer. After melt blending, the decomposition temperature of PLA/A 1.0 wt% was comparable to that of pure PLA, indicating that low plasticizer content does not significantly affect thermal stability. At 1.5 and 3.0 wt% ATEC, PLA blends exhibited a slight increase in decomposition temperature, with the highest Td observed for PLA/ATEC 1.5 wt%, indicating enhanced thermal resistance. This improvement is attributed to good miscibility at low-to-moderate plasticizer levels (≤1.5 wt%), which promotes molecular mobility. However, at higher ATEC contents (≥3.0 wt%), excess plasticizer may phase-separate or act as a less thermally stable component, accelerating degradation. Consequently, the blend containing 5.0 wt% ATEC showed reduced thermal stability compared to neat PLA. DSC analysis corroborated these results, revealing two melting peaks indicative of phase separation and diminished thermal integrity [23].

#### 3.1.4. Inherent Viscosity of PLA Blends with ATEC

To study changes in molecular weight of the polymer blends, inherent viscosities were measured and are shown in Figure 3e. PLA had an inherent viscosity of about 1.24 dL/g. PLA/A blends with 1.0–3.0 wt% ATEC showed slight increases in viscosity, indicating higher molecular weight and chain entanglement. However, at 5.0 wt% ATEC, a significant decrease in viscosity was observed, probably due to limited compatibility and interactions between PLA and the plasticizer at higher levels.

#### 3.1.5. Melt Flow Index of PLA Blends with ATEC

The melt flow index (MFI) results show that neat PLA has an MFI of about 9.4 g/10 min. The addition of 1.0% ATEC significantly increases the MFI to approximately 16.5 g/10 min, indicating improved chain mobility and melt flow. However, as ATEC content increases from 1.5 to 5.0 wt%, the MFI gradually decreases from approximately 14.0 g/10 min to around 8.8 g/10 min. This decrease at higher ATEC concentrations is attributed to the increased crystallinity seen in DSC analysis, which limits polymer chain mobility despite the plasticizing effect.

Based on the results, PLA blends with 1.0–5.0 wt% ATEC demonstrated optimal performance, balancing flexibility, thermal stability, and processability. Specifically, the PLA/ATEC 3.0 wt% blend (referred to as PLA/A) was selected for monofilament production, exhibiting increased tensile flexibility and molecular weight while maintaining tensile strength, with slight reductions in thermal transition temperatures compared to other blends. These combined properties make PLA/A 3.0 wt% an ideal candidate for further study.

### 3.2. Combined Effect of 3.0 wt% ATEC and 0.3 wt% TMC-200 on PLA

Subsequently, PLA and selected PLA-based blends—PLA/A (containing 3.0 wt% ATEC), PLA/T (containing 0.3 wt% TMC-200), and PLA/T/A (containing 3.0 wt% ATEC and 0.3 wt% TMC-200)—underwent an additional melt-blending step under the same processing conditions to improve their mechanical properties. This study further aimed to evaluate the effects of ATEC and TMC-200 on PLA, focusing on mechanical performance (tensile strength and elongation at break), thermal behavior, flow behavior, POM, and XRD. These assessments were crucial for understanding the impact of the plasticizer and nucleating agent on PLA’s properties and its suitability for FDM monofilament fabrication in medical applications (Section 3.3).

#### 3.2.1. Chemical Functionalities of PLA Blends with ATEC and TMC-200

**Figure 4:**
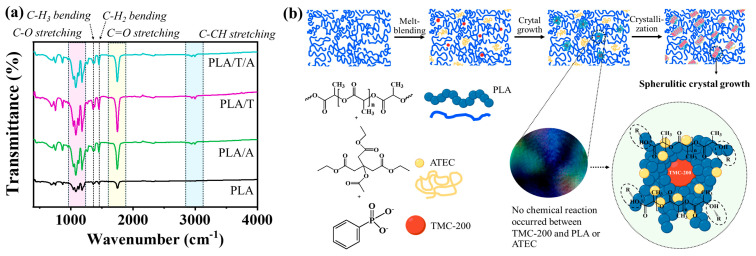
Structures and FTIR spectra of PLA, PLA/A, PLA/T, and PLA/T/A blends: (**a**) infrared spectra; (**b**) structural formula.

FTIR analysis (Figure 4) showed that all samples displayed characteristic absorption bands for C–O stretching, C=O stretching, and C–CH stretching, confirming the presence of PLA ester groups. In the case of PLA/T and PLA/T/A blends, the FTIR spectra indicated that the peak positions of the modified samples remained mostly unchanged, with no new peaks observed. This suggests that adding the nucleating agent TMC-200 did not alter the chemical structure of PLA [39].

#### 3.2.2. Mechanical Properties of PLA Blends with ATEC and TMC-200

**Figure 5:**
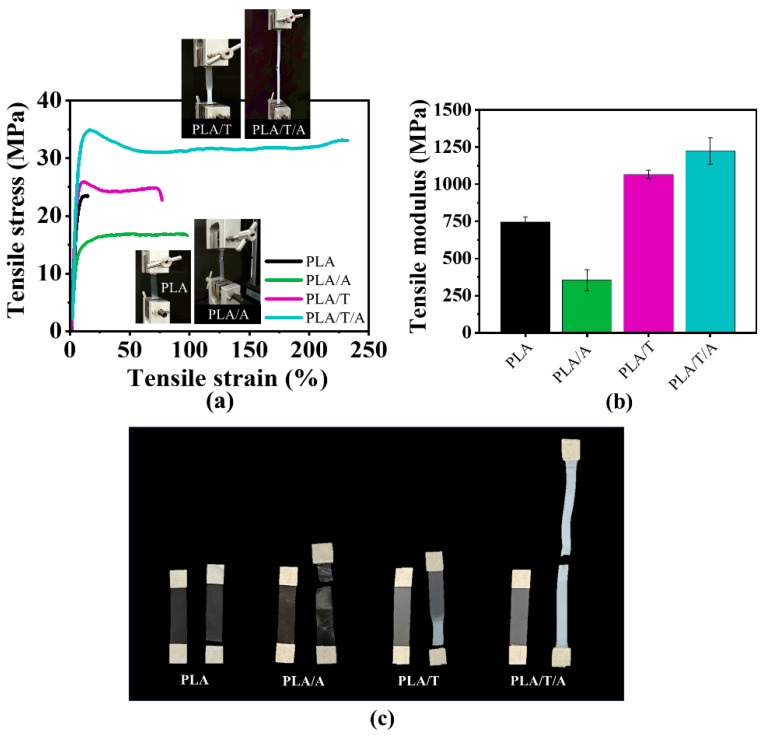
Mechanical properties of PLA, PLA/A, PLA/T, and PLA/T/A blends: (**a**) stress–strain curves; (**b**) tensile modulus; (**c**) comparison of tensile test samples before and after fracture.

The tensile properties of PLA, PLA/A, PLA/T, and PLA/T/A blends are shown in Figure 5a,b and Table 2. The result showed that pure PLA exhibited brittle fracture behavior, while PLA/A demonstrates improved ductility but reduced strength. With 0.3 wt% TMC-200 as a nucleating agent, the PLA/T blend showed improved strength (25.9 ± 1.5 MPa) and elongation (77.0 ± 0.1%) compared with PLA and PLA/A blends, indicating superior tensile performance and structural stability. These improvements can be attributed to crystallinity changes induced by the nucleating agent [30]. Meanwhile, the tensile strength, modulus, and elongation at break of the PLA/T/A ternary blend were notably higher than those of all other samples, indicating enhanced structural stability and a pronounced synergistic effect between ATEC and TMC-200 [40]. The fractured tensile samples further corroborated these results, showing that the PLA/T/A blends maintained good tensile strength while exhibiting markedly superior ductility compared to the other samples (Figure 5c).

#### 3.2.3. Thermal Properties of PLA Blends with ATEC and TMC-200

**Figure 6:**
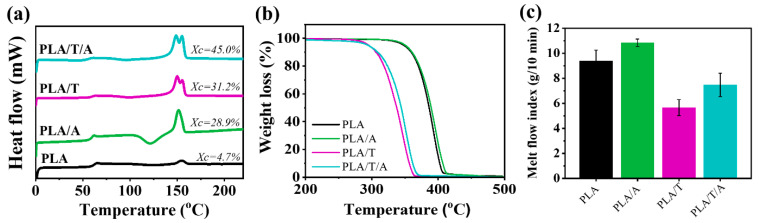
(**a**) DSC curve; (**b**) TGA curve; (**c**) melt flow index of PLA, PLA/A, PLA/T, and PLA/T/A blends.

Figure 6a shows the thermal properties of PLA, PLA/A, PLA/T, and PLA/T/A as analyzed by DSC and TGA. As previously noted, PLA displayed a T_g_ of 61.7 °C, a T_c_ of 126.7 °C, and a T_m_ of 154.7 °C, with the lowest degree of crystallinity (*X*_c_ = 4.68%), reflecting limited chain mobility and poor crystallization ability. In PLA/T, the incorporation of 0.3 wt% TMC-200 resulted in a decrease in the T_g_ and T_m_ to 59.6 °C and 150.0 °C, respectively. The crystallization peak temperature dropped significantly to 99.2 °C, which is 20–30 °C lower than that observed in PLA and PLA/A. Concurrently, the degree of crystallinity increased to 31.15%, demonstrating that the nucleating agent effectively enhanced the crystallization process [41]. In the case of PLA/T/A ternary blend, the thermal properties further decreased to 56.1 °C for T_g_, 96.2 °C for Tc, and 148.8 °C for Tm, mainly due to the plasticizing effect of ATEC, which enhances chain mobility. Meanwhile, TMC-200 provides heterogeneous nucleation sites that accelerate crystal formation. Together, these effects promote faster crystallization and the formation of finer, more uniform spherulite structures. These results demonstrate a synergistic effect of ATEC and TMC-200, which effectively enhances the crystallization behavior of PLA. Consequently, the degree of crystallinity increased significantly, with PLA/T/A exhibiting the highest *X*_c_ of 45.04% [30]. A similar trend was observed in a study of PLA/TMC/PEG blends, where the addition of TMC and PEG increased the degree of crystallinity while the T_g_ decreased due to the plasticizing effect of PEG [29]. Moreover, the addition of TMC-200 had two melting peaks. This is probably because TMC-200 promotes the formation of more and varied crystalline regions within PLA, leading to multiple melting points during heating [30,42].

As shown in Figure 6b, the TGA curves remained a single-step weight loss profile, leading to complete decomposition (100%), indicating good compatibility between PLA, the plasticizer, and the nucleating agent. TGA analysis revealed T_d(50%)_ values of 393.3 °C for PLA and 396.6 °C for PLA/A, while PLA/T and PLA/T/A blends exhibited lower values of 364.0 °C and 364.8 °C, respectively. The incorporation of TMC-200 decreased the thermal stability of PLA, which can be attributed to the residual hydroxyl groups present in TMC-200. These groups may promote hydrolysis of the PLA chains during melt processing, particularly in the presence of trace moisture [43]. Moreover, although TMC-200 acts as an effective nucleating agent that enhances the degree of crystallization, previous studies have shown that nucleation-induced crystallinity alone contributes less to heat resistance than lamellar thickening or structural perfection achieved through post-annealing, which strongly influences the crystallization morphology [41]. Therefore, while TMC-200 efficiently accelerates the crystallization of PLA, the overall thermal stability remains highly dependent on the nature of the increased crystallinity (e.g., crystallization morphology and degree of crystallinity) as well as moisture control during compounding [44]. In PLA/T/A, ATEC improves flexibility through plasticization, while TMC-200 promotes nucleation-driven crystallization, leading to slightly reduced thermal stability relative to PLA/T alone. Nevertheless, all samples retained sufficient thermal resistance up to approximately 300 °C.

#### 3.2.4. Melt Flow Index of PLA Blends with ATEC and TMC-200

As mentioned in Section 3.1.5, PLA exhibited an MFI of approximately 9.4 g/10 min, while the PLA/A blend showed the highest MFI (~11.0 g/10 min) due to the plasticizing effect of ATEC. In contrast, PLA/T displayed the lowest MFI (~5.7 g/10 min) due to the nucleating effect of TMC-200. The PLA/T/A blend exhibited an intermediate MFI (~7.5 g/10 min), reflecting a balance between enhanced chain mobility and restricted flow. These results demonstrate that melt processability can be effectively tuned by adjusting the contents of ATEC and TMC-200.

#### 3.2.5. Crystal Morphology of PLA Blends with ATEC and TMC-200

**Figure 7:**
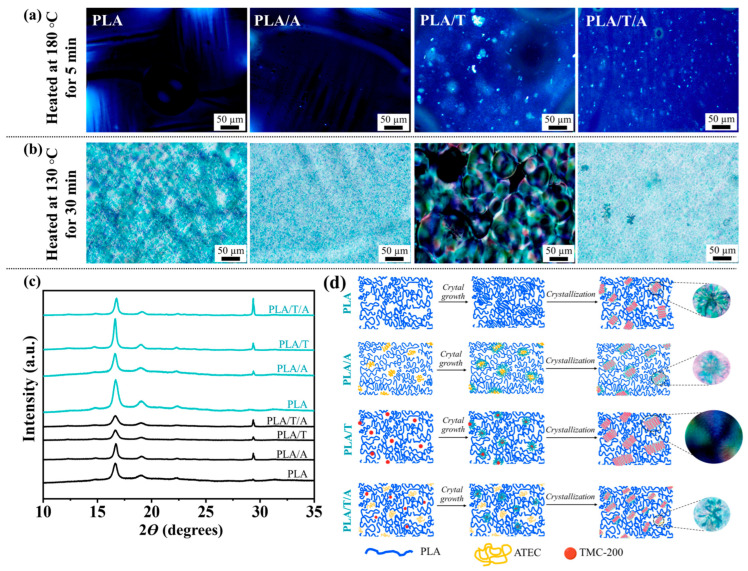
Crystal morphologies of PLA, PLA/A, PLA/T and PLA/T/A blends: (**a**) POM images after thermal treatment at 180 °C for 5 min; (**b**) POM images after thermal treatment at 130 °C for 30 min; (**c**) x-ray diffractograms of samples before (black) and after heat treatment at 130 °C for 30 min (blue); (**d**) schematic of the crystal morphology and after heat treatment at 130 °C for 30 min.

To further study the influence of TMC-200 on the crystallization behaviors of PLA, PLA/A, PLA/T, and PLA/T/A blends were studied using POM after thermal treatment at 180 °C for 5 min and 130 °C for 30 min (Figure 7a,b). It is obvious that pure PLA, a slow-crystallizing polymer, showed a relatively smooth and featureless morphology after treatment at 180 °C for 5 min, while crystallization was strongly suppressed at 130 °C for 30 min, resulting in a semi-crystalline structure. After the addition of ATEC, the spherulite size and number were reduced, resulting in less distinct crystalline structures due to enhanced chain mobility. Notably, the incorporation of TMC-200 (PLA/T) markedly enhanced nucleation, leading to the formation of many small crystallites at 180 °C and strong birefringence at 130 °C [41]. The ternary blend of PLA/T/A exhibited a more uniform distribution of smaller spherulites than PLA/T, reflecting the synergistic effect of TMC-200, which promotes nucleation, and ATEC, which enhances chain mobility. This balance results in improved crystallinity with smaller, evenly distributed spherulites, suggesting enhanced toughness and dimensional stability compared to the pure or binary blends [45].

#### 3.2.6. XRD of PLA Blends with ATEC and TMC-200

From the above investigations, it can be concluded that TMC-200 acts as an effective nucleating agent for PLA crystallization. To further support the POM observations, XRD analysis was performed [17,46]. As shown in Figure 7c, all blends exhibited the characteristic diffraction peaks of the α-form of PLA at around 16–17° and 18–19° (2θ), indicating that the additives did not alter the crystal form of PLA [47]. However, the sharpness and intensity of the peaks varied with composition and heat treatment. The diffraction peaks of both pure PLA and PLA/A were observed but appeared with relatively low intensity and broad profiles, reflecting their limited crystallinity. For pure PLA, this is attributed to its inherently slow crystallization kinetics, while in the case of PLA/A, the additional plasticizing effect of ATEC further reduces chain packing efficiency. Conversely, PLA/T already showed sharper peaks, which indicates that crystallization was improved by TMC-200 even before heat treatment. After annealing at 130 °C for 30 min (blue patterns), all samples exhibited an increase in peak intensity, indicating enhanced crystalline ordering. As a result, both PLA and PLA/A achieved a higher degree of crystallinity [38]. Specifically, the strongest peaks were observed in PLA/T, which matches the POM result of large and well-ordered spherulites. PLA/A displayed only a slight increase in crystallinity due to the plasticizing effect of ATEC, which hindered chain packing. Meanwhile, PLA/T/A showed crystallinity between PLA/A and PLA/T. This agrees with the POM images, where many small and uniform spherulites were seen instead of large and highly ordered ones [41,46].

#### 3.2.7. Rheology Testing of PLA Blends with ATEC and TMC-200

**Figure 8:**
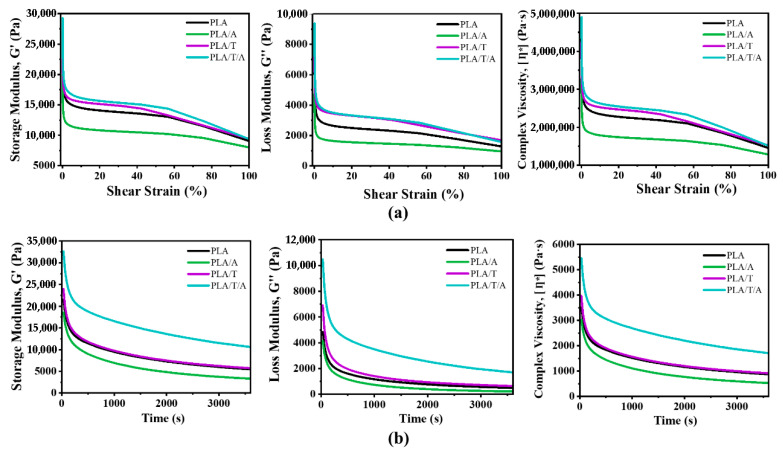
Rheological behavior of PLA, PLA/A, PLA/T and PLA/T/A blends: (**a**) strain sweep test showing storage modulus (G′) (left), loss modulus (G″) (middle) and complex viscosity (η*) (right) as a function of strain; (**b**) time sweep test showing the evolution of storage modulus (left), loss modulus (middle) and complex viscosity (right) over time under constant strain and frequency.

To further evaluate the flow behavior and viscoelastic stability of PLA, PLA/A, PLA/T, and PLA/T/A blends, two rheological tests were performed: a strain sweep and a time sweep (Figure 8). The strain sweep measured the material’s response to increasing strain at a constant frequency, whereas the time sweep monitored the stability of mechanical properties over time under constant conditions [48].

As shown in Figure 8a, the addition of ATEC to PLA (PLA/A) significantly decreased the storage modulus (G′), loss modulus (G″), and complex viscosity (η*) compared to PLA alone, consistent with enhanced chain mobility due to its plasticizing effect. On the one hand, PLA/T displayed higher G′, G″, and η* than both PLA and PLA/A, indicating the nucleating effect of TMC-200. The presence of a small amount of TMC-200 promoted PLA crystallization, thereby increasing rigidity and reducing molecular mobility [49]. Among all formulations, the PLA/T/A blend exhibited the highest G′, G″, and η* compared to PLA, PLA/A, and PLA/T, reflecting the combined effects of TMC-200–induced nucleation and ATEC-modulated chain mobility that together strengthen the network.

These trends were maintained in the time sweep measurements, where all samples maintained stable G′, G″, and η* over the test duration, confirming good structural stability. The result showed that PLA retained the highest values, whereas PLA/A maintained lower yet steady values, consistent with enhanced flowability (Figure 8b). PLA/T showed a gradual increase in modulus and viscosity over time, further indicating nucleation and progressive network formation induced by TMC-200. The PLA/T/A blend again exhibited intermediate behavior, demonstrating that the combined effects of ATEC softening and TMC-200 reinforcement produce a stable, balanced viscoelastic and flow response. Overall, these results indicate that the combination of additives can be used to tailor not only the initial rheological behavior but also the long-term stability of PLA-based systems during processing.

### 3.3. PLA/TMC-200/ATEC Blended Monofilaments

Hereafter, the effects of incorporating 3.0 wt% ATEC and 0.3 wt% TMC-200 into PLA monofilaments were evaluated in terms of surface morphology, mechanical properties, thermal behavior, and cytotoxicity. Furthermore, 3D-printed specimens fabricated from these monofilaments were examined to assess their printability, as well as their mechanical and thermal properties. Considering the limited reports on the influence of storage time and temperature on the quality of PLA and PLA/T/A monofilaments, their shelf life was further investigated. The evaluation was based on physical appearance and weight retention. Storage experiments were carried out at 50 °C, with assessments conducted after 0, 2, 4, and 8 weeks.

#### 3.3.1. Surface Morphological Analysis of PLA Monofilaments

It is important to first note the practical fabrication challenges encountered with unmodified PLA. The inherent brittleness of the pure PLA monofilament was a significant processing issue, causing it to frequently snap during spool winding and contributing to the nozzle clogging and cracking observed during initial 3D printing (discussed in Section 3.3.5). In contrast, the addition of ATEC and TMC-200 directly mitigated these problems. The enhanced ductility of the resulting PLA/T/A blend created a tough, flexible monofilament that could be extruded and spooled continuously without breakage, dramatically improving fabrication reliability, a critical prerequisite for FDM 3D printing.

**Figure 9:**
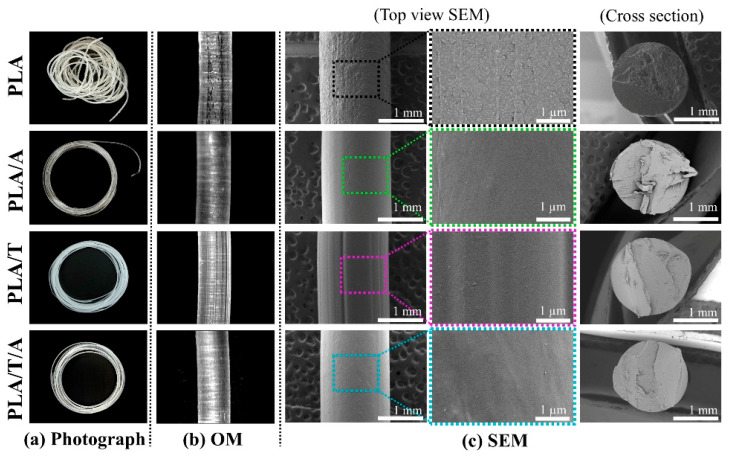
Surface morphology of PLA, PLA/A, PLA/T, and PLA/T/A monofilaments: (**a**) optical photographs; (**b**) optical microscopy (OM) images; (**c**) SEM images (left; top view from SEM at 40× magnification, middle; top view from SEM at 300× magnification (the zoomed images) and cross-section from SEM at 40× magnification).

The surface and cross-sectional morphologies of the PLA, PLA/A, PLA/T, and PLA/T/A monofilaments were examined using optical photography, optical microscopy (OM), and SEM (Figure 9). Optical images revealed that all monofilaments had diameters ranging from 1.70 to 1.79 mm, closely matching the optimal monofilament diameter for FDM 3D printing (1.75 ± 0.05 mm) (Figure 9a) [18]. The OM and SEM top-view images showed that the PLA monofilament exhibited a relatively rough surface, which can be attributed to the poor stability of PLA in the absence of stabilizing additives. As can be expected, the PLA/A monofilament displayed smoother and more uniform surfaces, reflecting the well-dispersed ATEC particles that enhance surface homogeneity by increasing polymer chain mobility and flow during extrusion [22,40,50,51]. Upon the addition of nucleating agents, PLA/T monofilament exhibited rougher surfaces with noticeable irregularities, reflecting microstructural heterogeneity from localized crystallization and restricted chain mobility induced by TMC-200; nevertheless, the overall surface remained relatively smooth [30]. The PLA/T/A monofilament exhibited intermediate surface features, demonstrating that the combination of ATEC and TMC-200 balances plasticization and nucleation effects to produce a more uniform surface. Cross-sectional images confirmed the monofilament diameters and revealed that the incorporation of additives affected the uniformity and homogeneity of the internal structure.

#### 3.3.2. Mechanical Properties of PLA Monofilaments

**Figure 10:**
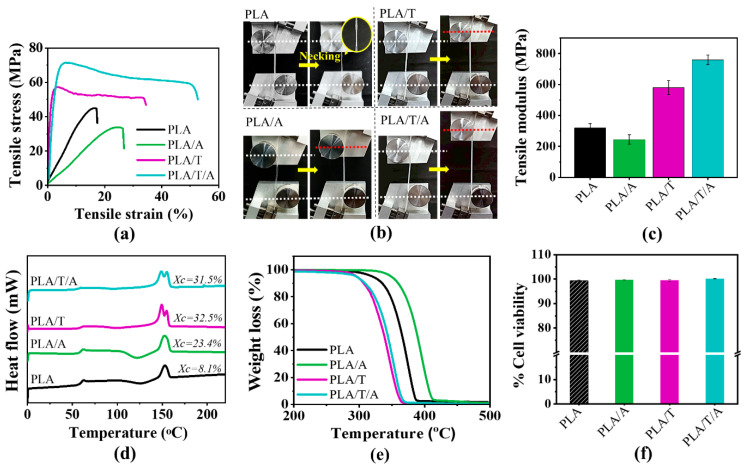
The properties of PLA, PLA/A, PLA/T, and PLA/T/A monofilaments: (**a**) tensile stress–strain curves; (**b**) photographs of samples before and after tensile testing; (**c**) tensile modulus; (**d**) DSC thermogram (second heating); (**e**) TGA thermogram; (**f**) indirect in vitro cytotoxicity test.

Figure 10a–c and Table 3 summarize the tensile properties of PLA, PLA/A, PLA/T, and PLA/T/A monofilaments. Apparently, pure PLA monofilament has relatively high tensile strength but breaks easily at low strain, showing its brittle nature with noticeable necking before failure. Adding ATEC (PLA/A) into PLA monofilament greatly reduces tensile strength but increases elongation, reflecting the plasticizing effect that enhances chain mobility and ductility. However, adding TMC-200 (PLA/T) promotes crystallization and increases rigidity, leading to improved tensile strength, modulus, and elongation compared to PLA and PLA/A. Notably, the ternary blend PLA/T/A monofilament shows the best overall performance, with significantly higher strength, modulus, and elongation, along with distinct strain-hardening behavior. Our recorded values for PLA/T/A monofilament (tensile strength ≥ 71 MPa and elongation at break ≥ 73%) are markedly higher than those reported in the literature for conventional PLA monofilaments, which typically exhibit tensile strengths of 50–70 MPa but much lower elongations at break, often below 10% [15,26,52,53,54]. As seen, it not only overcomes the inherent brittleness of standard PLA but also exhibits a mechanical profile that is highly competitive with, and in some aspects exceeds, established commercial-grade materials, making it a strong candidate for demanding biomedical applications.

Moreover, the photos in Figure 10b support these results, showing brittle fracture in PLA (indicated by necking in the yellow circle), more ductile deformation in PLA/A, and greater toughness and extensibility in PLA/T, with the strongest effect observed in PLA/T/A. Overall, combining ATEC and TMC-200 provides a synergistic improvement in the surface quality and mechanical properties of PLA-based monofilaments, achieving a balanced combination of strength and flexibility suitable for 3D printing.

#### 3.3.3. Thermal Performance of PLA Monofilaments

The PLA monofilament exhibited a T_g_ of 60.3 °C, T_c_ of 127.7 °C, and T_m_ of 153.8 °C, with a low crystallinity of 8.1% (Figure 10d and Table 3). Incorporation of ATEC slightly decreased T_g_ and T_m_, while markedly increasing crystallinity to 23.4%, reflecting enhanced chain mobility and improved molecular packing. Incorporation of the nucleating agent TMC-200 markedly reduced T_c_ to 99.2 °C and promoted the highest crystallinity (32.5%) by providing nucleation sites that facilitate accelerated crystal formation. When ATEC and TMC-200 were combined in PLA, the PLA/T/A monofilament exhibited a synergistic effect; ATEC enhanced chain flexibility, while TMC-200 promoted nucleation, leading to relatively high crystallinity (31.5%) and accelerated crystallization (T_c_ = 98.1 °C).

The thermal degradation of PLA monofilament occurred at T_d(50%)_ of 386.5 °C (Figure 10e and Table 3). As mentioned above, the addition of ATEC improved the thermal stability of PLA, increasing the T_d(50%)_ to 391.1 °C. In contrast, the incorporation of TMC-200 decreased the T_d(50%)_ to 353.4 °C, which can be attributed to differences in the resulting crystallinity—such as crystallization morphology and degree of crystallinity—as well as the influence of moisture control during compounding [43,44]. For the PLA/T/A monofilament, T_d(50%)_ was 358.2 °C, indicating that although the synergistic effect of ATEC and TMC-200 enhanced crystallization behavior, it slightly compromised thermal stability compared to PLA alone.

#### 3.3.4. Evaluation of Cytotoxicity of PLA Monofilaments

Cell cytotoxicity was evaluated through indirect contact according to ISO 10993-5:2009 guidelines [55,56]. The MTT assay was performed using mouse fibroblast L929 cells to assess the biocompatibility of PLA, PLA/A, PLA/T, and PLA/T/A monofilaments, as shown in Figure 10f. Instead of direct testing, the extraction media from the monofilament samples were applied to the cells. PLA exhibited a viability of 99.4%, while PLA/A and PLA/T showed 99.7% and 99.5%, respectively. The ternary blend PLA/T/A demonstrated the highest value at 100.2%. The results indicated that all monofilament samples were identified as nontoxic to fibroblast cells, since cell viability remained above 70% after all treatments. These results confirm that incorporating ATEC and TMC-200 into PLA monofilaments does not induce cytotoxicity in fibroblast cells, highlighting the importance of cytotoxicity testing in developing PLA-based monofilaments with excellent biocompatibility for biomedical applications.

This confirmed biocompatibility, coupled with the material’s superior mechanical properties and printability, makes the PLA/T/A monofilament a promising candidate for medical devices where structural integrity and resistance to brittle fracture are critical. While these initial cytotoxicity results are excellent, further validation is necessary to fully assess its potential for these applications. Therefore, future work will focus on more advanced in vitro assays, including cell proliferation studies and a comprehensive analysis of the material’s degradation rate and profile.

#### 3.3.5. Print Quality of the Printed Specimens

**Figure 11:**
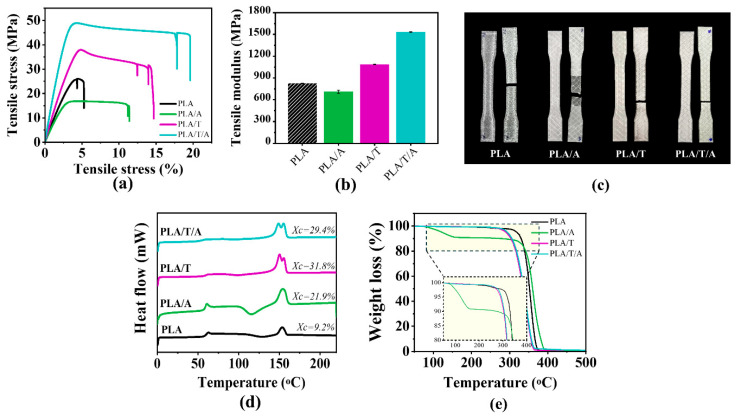
Properties of PLA, PLA/A, PLA/T, and PLA/T/A printed specimens: (**a**) tensile stress–strain curves; (**b**) tensile modulus; (**c**) photographs of specimens before and after tensile testing; (**d**) DSC thermograms (second heating); (**e**) TGA thermograms (zoomed in at 80–400 °C).

To evaluate the potential of the obtained monofilaments as candidates for FDM 3D printing, their printability was first assessed, followed by characterization of the mechanical and thermal properties of the printed specimens (Figure 11) [26]. The printing nozzle temperature was varied from 160 °C to 240 °C, while the layer infill and outer wall printing speeds were consistently maintained at 105 mm/s and 200 mm/s, respectively. The infill density was set to 15%.

The results showed that the printer was unable to consistently purge and extrude the monofilaments through the nozzle at 160–180 °C, leading to nozzle clogging and cracking during printing, which reflected the brittle nature of PLA. Therefore, the extrusion temperature was increased to 180–200 °C, at which continuous purging was achieved. At 200–220 °C, the specimens were successfully printed. PLA exhibited surface irregularities even at 220 °C, whereas PLA/A showed smooth extrusion and uniform surfaces at the same temperature. In the case of PLA/T and PLA/T/A, both demonstrated smooth extrusion and uniform surfaces, specifically at 210 °C. However, when the temperature was increased to 240 °C, the printer was able to extrude all monofilaments, but the printed specimens exhibited significant monofilament deformation and merging.

These qualitative improvements in printability are directly supported by the thermal and rheological properties of the material. The printability issues of pure PLA, such as nozzle clogging, are consistent with its lower melt flow index and higher complex viscosity (η*). Conversely, the smooth extrusion and uniform surfaces of the PLA/T/A blend are facilitated by its balanced MFI and stable rheological profile, ensuring consistent nozzle flow.

Furthermore, the crystallization behavior is critical to print quality. Pure PLA exhibited a high crystallization temperature (T_c_ of 127.7 °C) and very low crystallinity (8.1%) in the monofilament. This slow, uncontrolled solidification is a known cause of high internal stresses and differential shrinkage, which leads to warpage and poor interlayer bonding.

In contrast, the nucleating agent in our PLA/T and PLA/T/A blends induced a much lower and sharper T_c_ (around 98–99 °C) and significantly higher crystallinity (31.5–32.5%). This rapid crystallization promotes faster solidification and dimensional stability immediately after the material is deposited. This behavior is crucial for mitigating warpage and enhancing layer fusion, confirming the superior printability of the PLA/T/A formulation.

#### 3.3.6. Mechanical Performance of the Printed Specimens

Subsequently, the results of the tensile tests, which provide insight into the mechanical performance of the 3D-printed specimens, are summarized in Table 3 and illustrated in Figure 11a–c. As shown in Figure 11a,b, the incorporation of ATEC (PLA/A specimen) reduced tensile strength and modulus but markedly enhanced ductility due to its plasticizing effect. In contrast, the addition of TMC-200 (PLA/T specimen) increased both the tensile strength and modulus, while also improving the elongation at break compared to PLA alone. Remarkably, the combined addition of ATEC and TMC-200 (PLA/T/A specimen) produced a synergistic effect, resulting in the highest ultimate strength, modulus, and elongation at break. The recorded values for the PLA/T/A specimen (tensile strength ≥ 41 MPa and elongation at break ≥ 18%) were higher than those reported in the literature for standard 3D-printed PLA specimens, which typically exhibit tensile strengths of ≥30 MPa but much lower elongations at break, ranging from 2% to 10% [26,53,57].

Specimens with fractures showed clear differences in how they broke (Figure 11c). The PLA specimen broke suddenly with a clean fracture, which confirms its brittle nature. The PLA/A specimen had a more irregular and stretched fracture surface, matching its higher ductility from ATEC plasticization. However, the fracture surface also showed poor adhesion. The PLA/T specimen showed strain hardening and a more gradual break, reflecting the reinforcing and crystallization effect of TMC-200. For PLA/T/A, the specimens had the highest plastic deformation before breaking, marked by clear necking, elongated fracture regions, and better adhesion compared to PLA/A alone. This behavior shows a well-balanced mix of strength, stiffness, and ductility, resulting from the combined effect of ATEC and TMC-200. As a result, PLA/T/A overcomes the brittleness of pure PLA and offers a balanced mix of strength and flexibility suitable for advanced FDM 3D printing applications.

#### 3.3.7. Thermal Performance of the Printed Specimens

The thermal performance of the printed specimens was evaluated and compared to that of the monofilaments, with a particular focus on the effect of printing nozzle temperature. Therefore, DSC and TGA analyses were conducted to characterize their thermal properties. DSC results showed that T_g_, T_c_, and T_m_ remained unchanged after printing, with crystallization behavior similar to that of the monofilaments (Figure 11d). In the TGA analysis, it was observed that the T_d(50%)_ of PLA printed specimens decreased by approximately 5 °C, from 386.5 °C in the monofilaments to 381.3 °C after printing (Figure 11e). Notably, PLA/A exhibited a slightly earlier onset of degradation compared to its monofilament, indicating a minor reduction in thermal stability within the 160–330 °C range due to the incorporation of the plasticizer ATEC, with further degradation observed between 330 °C and 390 °C [58]. The thermal degradation behavior of PLA/T and PLA/T/A remained unchanged after printing, demonstrating consistency with their respective monofilaments. Overall, these results suggest that adding ATEC to PLA is effective for high-temperature applications, especially in FDM 3D printing. Although the combination of ATEC and TMC-200 slightly reduces PLA’s thermal stability, it still provides acceptable degradation resistance for FDM processing.

### 3.4. Estimation of Shelf Life of PLA and PLA/TMC-200/ATEC Monofilaments

#### 3.4.1. Physical Changes

**Figure 12:**
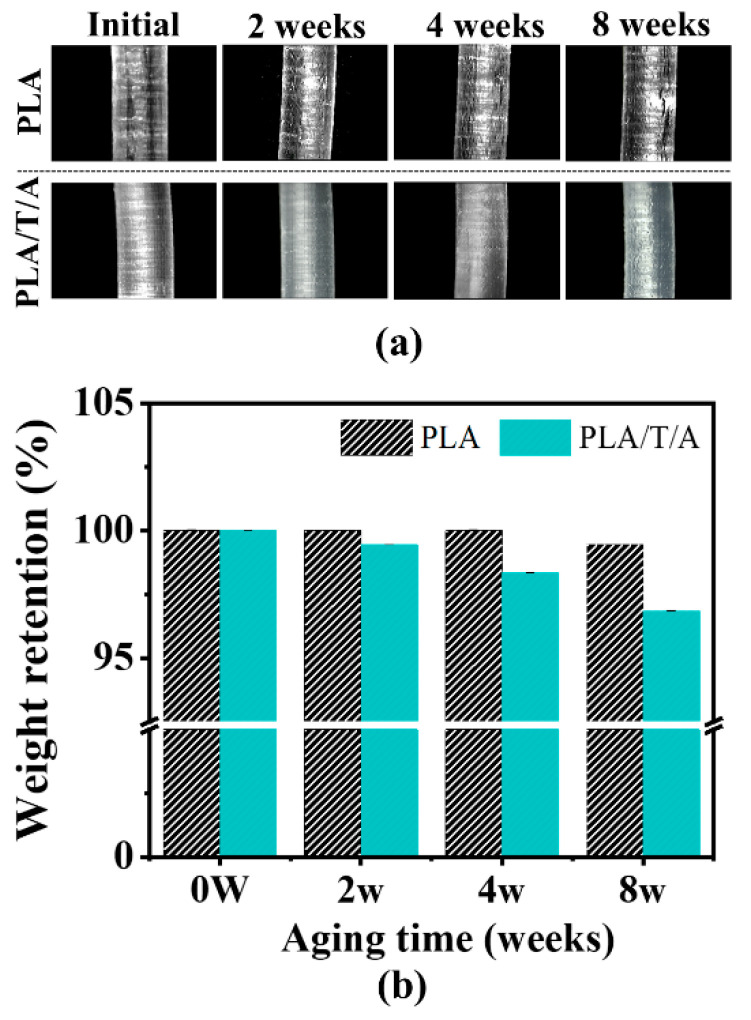
(**a**) Surface morphology; (**b**) weight retention of PLA and PLA/T/A monofilaments after thermal aging at 50 °C for 2 weeks, 4 weeks, and 8 weeks.

The optical microscopy images of PLA and PLA/T/A monofilaments shown in Figure 12a illustrate their surfaces after 2, 4, and 8 weeks at 50 °C from the fabrication date. Initially, both monofilaments exhibited smooth, uniform surfaces. After 8 weeks, PLA exhibited progressive surface roughening and microcrack formation with increasing storage time, indicating poor stability at elevated temperature. In contrast, PLA/T/A monofilaments showed relatively smoother and more uniform surfaces over the same period, although slight irregularities appeared after storage. This difference can be attributed to the combined effects of ATEC, which enhances chain mobility, and TMC-200, which promotes crystallinity, thereby reducing internal stress and improving structural stability.

#### 3.4.2. Weight Retention

Weight loss measurements before and after thermal aging were further studied and are presented in Figure 12b. The result revealed that PLA/T/A monofilament consistently exhibited higher degradation than PLA monofilament. After 8 weeks of storage, PLA monofilament retained over 99% of its weight, whereas PLA/T/A monofilament decreased to approximately 96%, indicating slightly lower but still acceptable resistance to thermal aging. While the presence of ATEC and TMC-200 contributed to maintaining surface integrity, the greater weight loss of PLA/T/A is likely attributed to the gradual migration of ATEC from the PLA monofilament matrix during prolonged storage [21,59].

## 4. Conclusions

This study investigated the impact of incorporating ATEC (as a plasticizer) and TMC-200 (as a nucleating agent) on the toughness of PLA monofilaments designed for medical-grade FDM 3D printing. PLA was first blended via reactive melt processing, with 3.0 wt% ATEC identified as the optimal concentration. This formulation improved PLA’s flexibility, although a slight reduction in tensile strength was noted compared to PLA alone. To enhance mechanical performance, PLA blends containing 3.0 wt% ATEC and 0.3 wt% TMC-200 were subsequently prepared. The results showed that the PLA/A blend containing the nucleating agent maintained a favorable balance between strength and toughness among all formulations, including PLA, PLA/A and PLA/T. As expected, ATEC primarily enhanced chain mobility and processability, whereas TMC-200 promoted crystallinity and mechanical reinforcement. Apparently, the PLA/T/A monofilaments were successfully fabricated with a smooth surface and a consistent diameter of 1.70 ± 0.05 mm. These monofilaments exhibited superior toughness and printability compared to pure PLA and binary blends. Additionally, cytotoxicity assessments demonstrated no cytotoxic effects, with over 99% cell viability, confirming that the PLA/T/A monofilaments are a highly promising candidate for biomedical applications. Thermal aging studies showed that PLA/T/A underwent slightly higher degradation than pure PLA but retained structural and surface integrity, confirming its reliable shelf-life and performance. Collectively, these findings highlight the potential of PLA/T/A as a next-generation, biocompatible filament for advanced FDM 3D printing applications.

## Figures and Tables

**Table 1:** Compositions of ATEC and TMC-200 additives used in the preparation of PLA-blended pellets by internal mixing.

Sample Codes	ATEC (A) (wt%)	TMC-200 (T) (wt%)
PLA	-	-
PLA/A 1.0%	1.0	-
PLA/A 1.5%	1.5	-
PLA/A 3.0%	3.0	-
PLA/A 5.0%	5.0	-
PLA/T	-	0.3
PLA/T/A	3.0	0.3

**Table 2:** Mechanical properties, thermal properties, and melt flow index of PLA blends.

Samples	Ultimate Strength (MPa)	Elongation at Break (%)	T_g_ (°C)	T_c_ (°C)	T_m_ (°C)	*X*_c_ (%)	T_d(50%)_ (°C)	MFI (g/10 min)
PLA	23.8 ± 0.2	12.1 ± 1.8	61.7	126.7	154.7	4.68	393.3	9.4 ± 0.9
PLA/A 1.0%	21.0 ± 1.4	26.6 ± 5.7	59.2	123.3	151.5	19.21	392.4	16.5 ± 0.9
PLA/A 1.5%	20.0 ± 1.1	42.2 ± 4.7	59.3	123.8	151.6	21.69	394.5	14.0 ± 0.6
PLA/A 3.0%	17.2 ± 0.3	99.0 ± 6.5	58.9	121.3	151.5	28.92	396.6	10.8 ± 0.3
PLA/A 5.0%	7.5 ± 0.5	336.1 ± 1.9	57.7	121.0	150.7	29.96	392.1	8.8 ± 0.6
PLA/T	25.9 ± 1.5	77.0 ± 0.1	59.6	99.2	150.0	31.20	364.0	5.7 ± 0.6
PLA/T/A	35.0 ± 0.3	232.0 ± 7.7	56.1	96.2	148.8	45.00	364.8	7.5 ± 0.9

**Table 3:** Mechanical and thermal properties of the extruded monofilaments and printed specimens.

Samples	Ultimate Strength (MPa)	Elongation at Break (%)	Tensile Modulus (MPa)	T_g_ (°C)	T_c_ (°C)	T_m_ (°C)	*X*_c_ (%)	T_d(50%)_ (°C)
Monofilaments								
PLA	45.3 ± 0.9	17.5 ± 0.9	319.8 ± 28.4	60.3	127.7	153.8	8.1	386.5
PLA/A	33.8 ± 5.2	27.0 ± 1.4	245.4 ± 29.7	58.3	121.7	152.5	23.4	391.1
PLA/T	57.3 ± 2.1	43.8 ± 4.1	579.5 ± 46.1	59.8	99.2	149.3	32.5	353.4
PLA/T/A	71.8 ± 3.1	73.2 ± 10.8	759.9 ± 30.6	57.3	98.1	148.8	31.5	358.2
3D printed specimens								
PLA	25.5 ± 0.4	5.1 ± 0.5	825.0 ± 4.5	60.2	128.2	153.5	9.2	381.3
PLA/A	17.3 ± 0.7	12.7 ± 2.5	711.3 ± 21.9	58.3	124.8	150.8	21.9	379.0
PLA/T	38.0 ± 1.1	13.7 ± 1.1	1088.3 ± 5.7	59.9	99.0	150.3	31.8	352.5
PLA/T/A	41.6 ± 0.3	18.4 ± 1.0	1534.2 ± 5.9	55.6	97.5	149.0	29.4	357.4

## Data Availability

The original contributions presented in this study are included in the article. Further inquiries can be directed to the corresponding author(s).
